# Formal verification of Matrix based MATLAB models using interactive theorem proving

**DOI:** 10.7717/peerj-cs.440

**Published:** 2021-03-22

**Authors:** Ayesha Gauhar, Adnan Rashid, Osman Hasan, João Bispo, João M.P. Cardoso

**Affiliations:** 1 School of Electrical Engineering and Computer Science (SEECS), National University of Sciences and Technology (NUST), Islamabad, Pakistan; 2 Faculty of Engineering, University of Porto, Porto, Portugal

**Keywords:** MATLAB, Formal verification, Matrix based MATLAB models, Interactive theorem proving, HOL Light, Formal Methods, Higher-order logic

## Abstract

MATLAB is a software based analysis environment that supports a high-level programing language and is widely used to model and analyze systems in various domains of engineering and sciences. Traditionally, the analysis of MATLAB models is done using simulation and debugging/testing frameworks. These methods provide limited coverage due to their inherent incompleteness. Formal verification can overcome these limitations, but developing the formal models of the underlying MATLAB models is a very challenging and time-consuming task, especially in the case of higher-order-logic models. To facilitate this process, we present a library of higher-order-logic functions corresponding to the commonly used matrix functions of MATLAB as well as a translator that allows automatic conversion of MATLAB models to higher-order logic. The formal models can then be formally verified in an interactive theorem prover. For illustrating the usefulness of the proposed library and approach, we present the formal analysis of a Finite Impulse Response (FIR) filter, which is quite commonly used in digital signal processing applications, within the sound core of the HOL Light theorem prover.

## Introduction

MATLAB (http://www.mathworks.com/products/matlab/) (MATrix-LABoratory) is arguably one of the most commonly used software environments for modeling and analyzing complex systems in various domains of engineering and science, including analog and mixed signal circuits, digital filters and control systems. One of the prime motivations of its widespread usage is the availability of a collection of built-in functions based on basic matrix operations, which can be built upon for developing a library of larger, more complex, functions.

Generally, model-based systems are represented in Simulink (http://www.mathworks.com/products/simulink/), while algorithms are expressed using *m*-code in MATLAB based analysis of systems. Traditionally, the Simulink models are validated through simulation, and the *m*-code based models are analyzed through debugging, that is, by setting breakpoints at different levels and examining the values of outputs/variables. Some testing frameworks (https://www.mathworks.com/help/matlab/matlab-unit-test-framework.html) are also used to partially test the MATLAB code by providing a subset of all possible input combinations.

Simulation and testing do not guarantee a complete analysis, since the system under test is simulated for a specific set of inputs and at specified intervals of time. Similarly, the breakpoints based approach tests the behavior of the given program by inserting breakpoints at various discrete steps only. It is quite feasible when dealing with smaller systems but becomes unscalable for larger systems, as managing breakpoints and deciding their suitable positions to ensure that the whole range of possible values is covered becomes extremely difficult. The use of computer arithmetic and thus the introduction of roundoff errors adds another dimension of inaccuracy to the MATLAB based analysis. Due to these inaccuracies in the analysis, the traditional MATLAB based analysis is not suitable for analyzing systems used in safety-critical domains, such as health-care or transportation, where even a slight inaccuracy or a missed corner case may lead to disastrous consequences including the loss of human life.

The above-mentioned limitations can be addressed by using formal methods (Hasan & Tahar, 2015) for analyzing MATLAB models. With the same motivation, MATLAB has recently introduced a formal verification tool, namely Simulink Design Verifier (SDV) (Hamon, 2008), to verify Simulink models. SDV supports two modes of operation: test suite generation and property proving. The first mode deals with the generation of suitable input vectors to provide the maximum output coverage and diversity for detecting bugs. The second mode is based on automated theorem proving and model checking techniques and is used for verifying the given system and generating counter examples in case of failures. However, this verification support is limited to a subset of Simulink functions and SDV has been reported to have some compatibility issues for larger models due to their nonlinear operations (Hamon, 2008). Even though the vector generation mode of SDV has been made quite scalable recently (Matinnejad et al., 2016), only a subset of blocks can be supported by the verification mode of SDV. Finally, due to the computational limitations of automatic theorem proving and model checking, the verification support of SDV cannot be utilized to verify physical systems that usually contain many continuous elements.

Another notable formal verification approach for Simulink models is presented in Chen, Dong & Sun (2009), where the main idea is to translate the Simulink models to a library of Timed Interval Calculus (TIC) functions, and then formally verify these TIC models. A library of TIC models is developed for the Simulink library blocks and each block can thus be automatically translated to its corresponding TIC model. Furthermore, the higher-order-logic theorem prover PVS (Owre, Rushby & Shankar, 1992) is used to formally verify these TIC models. These TIC models capture the timing characteristics of the given system and thus can provide the support for verifying the continuous models of systems by leveraging upon the high expressiveness of higher-order logic. However, the scope of their work is just limited to the Simulink models. The authors in Lu & Mukhopadhyay (2012) proposed using static code analysis, that is, to check the logical behavior of the code at compile time with abstract interpretation techniques to validate the *m*-code. However, their approach only ensures the verification of logical errors and cannot be used to capture the continuous aspects of the systems in their true sense.

Reicherdt & Glesner (2014) proposed to conduct the formal verification of the discrete-time MATLAB/Simulink models using Microsoft’s Boogie program verifier. It involves the automatic translation of MATLAB/Simulink models into the Boogie verification language, which allows developing first-order-logic based models of the system and their verification using the automated theorem prover Z3. Similarly, Boström (2011) proposed an approach for the contract-based verification of the Simulink models. It involves translating the Simulink models, viewed as Synchronous Data Flow (SDF) graphs, to their corresponding functionally equivalent sequential program statements, which are further analyzed using the automated theorem prover Z3. Joshi & Heimdahl (2005) proposed an approach for the verification of the discrete time MATLAB/Simulink models using the SCADE design verifier. It includes the translation of the MATLAB/Simulink models to the synchronous data flow language, Lustre, for the model-based safety analysis using SCADE. However, due to the inherent computational limitations of the associated formal methods, that is, less-expressiveness and abstraction in automated theorem proving, and discretization of the continuous models and state-space explosion in model checking, the above-mentioned approaches cannot be termed as accurate and complete when analyzing the complex systems exhibiting continuous dynamics.

Formalizations of real vectors and real matrices have been proposed in higher-order-logic theorem provers HOL Light (Harrison, 2013), HOL4 (Shi et al., 2014; Shi, Guan & Li, 2020), PVS (Herencia-Zapana et al., 2012), Isabelle (Aransay & Divasón, 2015), Mizar (Bancerek et al., 2018) and Coq (Boldo, Lelay & Melquiond, 2015; Mahboubi & Tassi, 2017; Dénes & Bertot, 2011) and automated theorem prover ACL2 (Gamboa, Cowles & Baalen, 2003). Also, the complex vectors, bivectors and some complex matrix arithmetic is formalized in HOL Light (Afshar et al., 2014). Similarly, these real vectors libraries have been used for formally verifying control software algorithms (Herencia-Zapana et al., 2012) and motion planner of autonomous vehicles (Rizaldi et al., 2018). However, these works mainly emphasize on the formalization of vectors and matrices, and not on matrix manipulation functions, such as, concatenation and flip operations, and thus require a significant amount of formalization effort for analyzing the applications in which a large number of matrix manipulations are required.

To overcome the above-mentioned limitations, we propose to formalize some of the most commonly used matrix functions, such as concatenation, flip, sum and product, of MATLAB in HOL Light (Harrison, 2009), which is a higher-order-logic theorem prover. The usage of higher-order logic and interactive theorem proving in the proposed analysis method allows us to overcome the expressiveness and scalability issues of SDV (Hamon, 2008) by leveraging upon the expressiveness of higher-order logic and the associated powerful reasoning methods, such as induction. Moreover, the inherent soundness of theorem proving ensures a complete and accurate analysis. It is important to note that these added benefits are attained at the cost of extensive human involvement in the formalization and verification tasks. The proposed work mainly focuses on the analysis of the MATLAB models expressed using the *m*-code. However it can also be used to analyze the Simulink models (Chen, Dong & Sun, 2009) since *m*-code can also be used to model Simulink blocks.

The main motivation of developing the proposed library is to facilitate the formal modeling process as these formal functions can be readily built upon to develop more complex formal models, just like we build complex models based on the available MATLAB functions. Moreover, we have also developed a translator that can parse a given MATLAB code and then utilize our formal library of MATLAB matrix functions to automatically develop the corresponding formal model in higher-order-logic. Thereafter, our formally verified properties of the proposed matrix functions can be used in the formal reasoning process of the given system and thus their availability reduces the interactive verification effort. In order to illustrate the practical effectiveness and utilization of the proposed formalization, we use it in this article to conduct the formal analysis of a Finite Impulse Response (FIR) filter that is quite frequently used in many Digital Signal Processing (DSP) applications.

The main contributions of this article are:

- Development of a formal library of some commonly used MATLAB functions in higher-order logic
- Formal verification of some of the classical properties of the formalized MATLAB functions using the HOL Light theorem prover
- Formal analysis of a Finite Impulse Response (FIR) filter using the proposed framework in HOL Light

## Multivariate theory in hol light

HOL Light (Harrison, 2009) is an interactive theorem prover from the family of Higher-order-logic (HOL) theorem provers. Its core primarily consists of a small number of basic axioms and inference rules, expressed in the Objective CAML (OCaml) language, which is a variant of the strongly typed functional language ML (*meta-language*) (Rémy, 2002). HOL Light has a rich set of theories for different data types, like natural numbers, real numbers, sets, lists, vectors, matrices etc. Despite having the basic formalization of the matrices and some of their operations in other theorem provers, the main motivation of choosing HOL Light for our work is its rich formal reasoning support for multivariate calculus (Harrison, 2013). One of the other reasons for opting HOL Light is the presence of formal libraries for the Laplace (Taqdees & Hasan, 2013; Rashid & Hasan, 2017) and the *z* (Siddique, Mahmoud & Tahar, 2014) transforms that allows us to link our proposed formalization of matrix functions to these libraries for performing the analysis of the continuous and discrete time systems, respectively.

We inherited the representation of an *M × N* matrix A as (**A**:real^N^M) from Harrison’s seminal work (Harrison, 2005). The idea here is that instead of defining with a specific type of matrices, the *M × N* matrices are represented using the Cartesian product twice and thus, the arithmetic operations can in turn be defined by using a point-wise lifting. Due to this formalization approach, we make the indexing correspond to the usual row-column convention by representing *M × N* matrices as (R^*N*^)^*M*^. Lambda abstraction function lambda is used to define the behavior of a vector or a matrix in terms of its components. Some of the HOL Light functions, from Harrison’s matrix theory (Harrison, 2005), used in this article are described below:

**Definition 1.** Matrix Multiplication

⊦*_def_* ∀(A:real^N^M) (B:real^P^N). *matrix_mul* A B = lambda i j. \sum^N_{k=1} A_ik_ ∗ B_kj_

The function matrix_mul accepts matrices A and B having dimensions *M × N* and *N × P*, respectively, and returns their multiplication result.

**Definition 2.** Transpose of a matrix

⊦_*def*_ ∀A. *transp* A = lambda i j. A_ji_

The function transp returns the transpose of the input matrix of order *M × N* by interchanging its rows and columns.

**Definition 3.** Row and Column of the Matrix

⊦_*def*_ ∀i A. *row* i A = lambda j. A_ij_

⊦_*def*_ ∀j A. *column* j A = lambda i. A_ij_

The function row accepts a natural number i and an *M × N* matrix A and extracts the *i*th row of the input matrix. The function column returns the *j*th column of the input matrix A.

**Definition 4.** Diagonal Matrix

⊦_*def*_ ∀k. *mat* k = lambda i j. if i = j then k else 0

The function mat accepts a natural number k and returns the diagonal matrix of order *M × N* with all the diagonal elements equal to k (Harrison, 2005). Thus, *k* = 1 gives an Identity matrix and *k* = 0 corresponds to the zero matrix. The & operator is used to typecast a natural number to its corresponding real number.

**Definition 5.** Vector

⊦_*def*_ ∀n. *vec* n = lambda i. &n

The function vec takes a natural number n and returns the vector with all its elements equal to n after changing it to a real number.

**Definition 6.** Row and Column Vectors

⊦_*def*_ ∀v. *rowvector* v = lambda i j. v_j_

⊦_*def*_ ∀v. *columnvector* v = lambda i j. v_i_

The functions rowvector and columnvector accept an *N*-dimensional vector v and return the same vector as the row and column matrices of orders *1 × N* and *N × 1*, respectively.

**Definition 7.** Vectorize

⊦_*def*_ ∀A. *vectorize* A = lambda i. A}{}_{(1+\frac{i-1}{N})(1+|i-1|_N)}

The function vectorize linearizes the input matrix A with order *M × N* to a (*M∗N*)-dimensional vector. Here, |x|_N_ represents the modulo operator, that is, it is a remainder after the division of x by N.

**Definition 8.** Matrify

⊦_*def*_ ∀(x:real^(M,N):finite_prod). *matrify* x = lambda i j. x_((*i* − 1)∗*N* + *j*)_

The function matrify takes a (*M*∗*N*)-dimensional vector x and returns the matrix with the order *M × N* having the same elements as the input vector. Here, finite_prod is a type constructor, which accepts two finite natural numbers M and N and returns the type *M × N*.

## Formalization of matrix functions in hol light

This section provides the proposed formalization of the commonly used matrix functions of MATLAB in HOL Light (Harrison, 2009). These formal definitions can in turn be used to convert a MATLAB model into its corresponding higher-order-logic model. It is important to note that the MATLAB functions use the IEEE floating point data type for the matrix elements due to the inability of expressing pure real numbers in computers. On the other hand, we have used the data type of real numbers for the elements of matrices in the corresponding formal definitions because the formal analysis is done symbolically with no involvement for computer arithmetic. Moreover, under certain circumstances, floating point numbers can be considered a subset of real numbers (Harrison, 2012). Finally, this is how we can deal with pure continuous models as well that MATLAB based analysis cannot handle.

We formalized most of the matrix manipulation functions that fall under the category of MATLAB language fundamentals (https://www.mathworks.com/help/matlab/functionlist.html). We describe some of the MATLAB functions that we have formalized in detail below, some are summarized in Table 1 and the rest can be found in (Gauhar, 2021).

**Table 1:** Formal definitions of MATLAB’s matrix manipulation functions.

Description	HOL Definition
horzcat (https://www.mathworks.com/help/matlab/ref/horzcat.html)	
Concatenates the input matrices horizontally	⊦_*def*_ ∀ (A:real^N^M) (B:real^P^M). *horz_conct* A B = lambda i j. if j = N then A_ij_ else B_i(j-N)_
vertcat (https://www.mathworks.com/help/matlab/ref/vertcat.html)	
Concatenates the input matrices vertically	⊦_*def*_ ∀ (A:real^N^M) (B:real^N^P). *vert_conct* A B = lambda i j. if i = M then A_ij_ else B_(i-M)j_
flipud (https://www.mathworks.com/help/matlab/ref/flipud.html)	
Flips the matrix along the horizontal axis	⊦*_def_* ∀(A:real^N^M) . *flipud* A = lambda i j. A_(M−i+1) j_
fliplr (https://www.mathworks.com/help/matlab/ref/fliplr.html)	
Flips the matrix along the vertical axis.	⊦*_def_* ∀(A:real^N^M) . *fliplr* A = lambda i j. A_i(N−j+1)_
movprod (https://www.mathworks.com/help/matlab/ref/movprod.html)	
Computes the product of k neighboring elements of input matrix A for each element of the output matrix	⊦*_def_* ∀(A:real^N^M) k. *movprod* A k = lambda i j. *if* |k|_2_ = 0 *then* \Pi^{i+(\frac{k}{2})}_{x=i-(\frac{k}{1})} (*if* *x* = 0 \vee *x* > *M* *then* 1 *else* A_xj_) *else* \Pi^{i + (\frac{k-1}{2})}_{x = i- (\frac{k-1}{2})} (if *x* = 0 \vee *x* > *M* *then* 1 *else* A_xj_)
Computes the product of previous k_1_ elements, next k_2_ elements and the current element	⊦*_def_* ∀(k:num^2^1) (A:real^N^M) k. *movprod* A k = lambda i j. \Pi^{i+k_2}_{i-k_1} (if x = 0 \vee x > M *then* 1 *else* A_xj_)
cumprod (https://www.mathworks.com/help/matlab/ref/cumprod.html)	
Cumulative product of elements along rows of the given matrix	⊦*_def_* ∀A. *cumprod_row* A = lambda i j. \Pi^{j}_{k = 1} A_ik_
Cumulative product of elements along columns of the given matrix	⊦*_def_* ∀A. *cumprod_col* A = lambda i j. \Pi^{j}_{k = 1} A_kj_
cumsum (https://www.mathworks.com/help/matlab/ref/cumsum.html)	
Cumulative sum along each row	⊦*_def_* ∀A. *cumsum_row* A = lambda i j. \sum^{j}_{k = 1} A_ik_
Cumulative sum along each column	⊦*_def_* ∀A. *cumsum_col* A = lambda i j. \sum^{j}_{k = 1} A_kj_
sum (https://www.mathworks.com/help/matlab/ref/sum.html)	
Adds the elements of each column	⊦*_def_* ∀(A:real^N^M). *sum_row* A = lambda j. \sum^{M}_{k = 1} A_kj_
Adds the elements of each row to return a column vector	⊦*_def_* ∀(A:real^N^M). *sum_col* A = lambda i. \sum^{N}_{k = 1} A_ik_
prod (https://www.mathworks.com/help/matlab/ref/prod.html)	
Multiplies the elements of each column	⊦*_def_* ∀(A:real^N^M). *prod_row* A = lambda j. \Pi^{M}_{k = 1} A_kj_
Multiplies the elements of each row and returns a column vector	⊦*_def_* ∀(A:real^N^M). *prod_col* A = lambda i. \Pi^{M}_{k = 1} A_ik_
size (https://www.mathworks.com/help/matlab/ref/size.html)	
Returns the size of *M × N* input matrix A	⊦*_def_* ∀(A:real^N^M). *size_matrix* A = (M,N)
numel (https://www.mathworks.com/help/matlab/ref/numel.html)	
Returns the number of elements of the matrix A	⊦*_def_* ∀(A:real^N^M). *numel_mat* A = (M ∗ N)
Returns the number of elements for *N*-dimensional vector v	⊦*_def_* ∀(A:real^N). *numel_vect* v = N
length (https://www.mathworks.com/help/matlab/ref/length.html)	
Returns the maximum dimension for the matrix A	⊦*_def_ ∀*(A:real^N^M). *length_mat* A = max (M,N)
Returns the dimension of input vector v	⊦*_def_ ∀*(A:real^N). *length_vect* v = N
colon (https://www.mathworks.com/help/matlab/ref/colon.html)	
Returns the range from the lower limit l to upper limit u	⊦*_def_* ∀x u l. *colon1* x u l = (x ≤ u) \wedge (l ≤ x)
Returns the range from lower limit l to upper limit u for the variable x of natural number type that is, for the for-loop indexing	⊦*_def_* ∀x l u. *colon2* x l u = (x ≤ u) \wedge (l ≤ x)
Breaks the interval according to the given step size s	⊦*_def_* ∀x l u s n. *colon3* x l u s n = if l + ns ≤ u then x = l + ns else x = u
Times(.∗) (https://www.mathworks.com/help/matlab/ref/times.html)	
Element-wise multiplication	⊦*_def_* ∀A B. *array_mul* A B = lambda i j. A_ij_ ∗ B_ij_
Power(.^) (https://www.mathworks.com/help/matlab/ref/power.html)	
Power of each element of matrix A raises to k	⊦*_def_* ∀A k. *matpow* A k = lambda i j. A_ij_^k^
abs (https://www.mathworks.com/help/matlab/ref/abs.html)	
Returns absolute value of each element in matrix	⊦*_def_* ∀A. *abs_matrix* A = lambda i j. abs (A_ij_)
ind2sub (https://www.mathworks.com/help/matlab/ref/ind2sub.html)	
Changes the linear index i to the subscripts	⊦*_def_* ∀(A:real^N^M) i. *ind2sub* A i = }{}(|i - 1|M,\; {\frac {i-1}\over{M}})
sub2ind (https://www.mathworks.com/help/matlab/ref/sub2ind.html)	
Returns the index for given subscripts	⊦*_def_* ∀(A:real^N^M) i j. *sub2ind* A i j = (i + (j − 1) ∗ M)
rot90 (https://www.mathworks.com/help/matlab/ref/rot90.html)	
Rotates the input matrix by 90° in counter-clockwise direction	⊦*_def_* ∀A. *rot90_matrix_single* A = flipud (transp A)

### Rotation

*MATLAB function: rot90* (https://www.mathworks.com/help/matlab/ref/rot90.html)

*Behavior:* Rotates the contents of the input matrix A by integer multiples of 90° in the counter-clockwise direction.

**Definition 9.** Rotation by Multiples of 90°

⊦_def_ ∀A n. *rot90_matrix* A 0 = A ∧

*rot90_matrix* A (n + 1) = flipud (transp (*rot90_matrix* A n))

Our formalization of the rotation function rot90_matrix accepts a matrix A and a natural number n and rotates the matrix A in the counter-clockwise recursively by n ∗ 90 degrees by taking the transpose (transp) of the given matrix and then flipping it upside down (flipud) n times. The transp function, as described in “Multivariate Theory in HOL Light”, is a built-in HOL Light function whereas we formalized flipud as part of this work and its definition is in Table 1.

### Diagonalization

*MATLAB function: diag* (https://www.mathworks.com/help/matlab/ref/diag.html)

*Behavior:* The *diag* function can be used with a different number of input arguments. When there is a single input, that is, a matrix, it returns the main diagonal of input matrix A as a vector. For two input arguments, a matrix A and an integer k, it extracts the *k*th diagonal of the matrix A. For two inputs, a vector v and an integer k, it places the vector v in the *k*th diagonal, where *k* = 0 corresponds to placement of the vector in the main diagonal, and *k* < 0 and *k* > 0 are below and above the main diagonal, respectively.

**Definition 10.** Diagonal of a Matrix

⊦_def_ ∀A. *diag_single* A = lambda i. A_ii_

⊦_def_ ∀A k. *diag_vect* A k = lambda i. if k *≤* 0 then A_(i + k)i_ else A_i(i + k)_

⊦_def_ ∀v k. *diag_matrix* v k = lambda i j. if k *≤* 0 *then* (*if* i = j + k *then* v_j_ *else* 0)

                    *else* (*if* i + k = j *then* v_i_ *else* 0)

The formalization of the function *diag* is done using three definitions: diag_single, diag_vect and diag_matrix. The function diag_single accepts the matrix A and returns its main diagonal as a vector. To extract the *k*th diagonal of the input matrix A, diag_vect can be used. The function diag_matrix places input vector v in the *k*th diagonal of output matrix.

### Circular shift

*MATLAB function: circshift* (https://www.mathworks.com/help/matlab/ref/circshift.html)

*Behavior:* The function *circshift* has some variants depending upon the types of input arguments. circshift(A,[k_1_,k_2_]) shifts the matrix A in each dimension circularly, where k_1_ and k_2_ are the circular shift in row and column dimensions, respectively. circshift(v,k) circularly shifts the elements of the given vector v on the *k*th position, *k* > 0 and *k ≤* 0 corresponds to the right and left shift, respectively.

**Definition 11.** Circular Shifting of a Matrix

⊦_*def*_ ∀(v:real^M) k. *circ_vshift* v k = lambda n. *if |*i − k|_M_ *≤* 0 *then* v_(M − }{}|i - k|_M)_ else v_*}{}|i-k|_M*_

⊦_*def*_ ∀(A:real^N^M) k. *circ_mshift* A k =

          lambda i j. if (*|i* − k_1_*|*_M_ *≤* 0 ∧ *|*j − k_2_*|*_N_ *≤* 0) *then* A_(*M −*}{}|i - k_1|_M) (*N −*}{}| j - k_2|_N)_

                    *else if* (*|i − k*_1_*|_M_ ≤* 0) *then* A_(*M −*}{}|i - k_1|_M)(}{}|j - k_2|_N)_

                         *else if* }{}|j - k_2|_N *≤* 0 then A_(}{}|i - k_1|_M)(}{}|j - k_2|_N)_ *else* A_(}{}|i - k_1|_M)(}{}|j - k_2|_N)_

We formalized the behavior of the circular shift for matrices and vectors in two separate definitions, circ_mshift and circ_vshift, respectively. The circular shift in both cases is done by placing the corresponding entries of input vector/matrix using the if-then-else statements. The computation of corresponding indices involves the mod operator and some other arithmetic manipulations using the sizes/dimensions of the input vector/matrix and the index of the output vector/matrix.

### Colon indexing

*MATLAB function: Colon (:)* (https://www.mathworks.com/help/matlab/ref/colon.html)

*Behavior:* Colon (:) operators allow accessing the elements of a matrix. For example, a range of elements in matrix A can be accessed either through A(l:u) that returns (*u* − *l* + 1) consecutive elements starting from the lower limit l or through A(l:k:u) that returns \big(\frac{(u-l)}{k}{+1}\big) elements starting from the lower limit l until the upper limit u, while picking elements after every (k − 1) indices.

**Definition 12.** Matrix Indexing using the Colon Operator

⊦_def_ ∀A l u. *mat_colon* A l u = lambda i j.

          *if* l *≤* u *then* (*if* l + (*j* − 1) *≤* u *then* A_ind2sub(*A*)(*l* + (*j −* 1))_ *else* 0)

               *else* (*if* *u* + (*j* − 1) *≤* (l + 1) *then* A_ind2sub(*A*)((*l* + 1) *− j*))_ *else* 0)

⊦_def_ ∀A l k u. *mat_colon_interval* A l k u = lambda i j.

          *if* l *≤* u *then* (*if* *j ≤* \frac{(u-l)}{k} *then* A_ind2sub(*A*)(*l* + *k*∗(*j −* 1))_ *else* 0)

               *else* (*if* *j ≤* \frac{(u-l)}{(k+1)} *then* A_ind2sub(*A*)(*l − k∗*(*j −* 1))_ else 0)

The return type of colon indexing functions depends on the value of the input. However, this behavior cannot be formalized in HOL Light as we need to explicitly declare the return type of the functions in their function definition. To cater for this issue, we decided to keep the dimensions of the return type of our colon indexing matrices the same as the input matrices by adding additional 0s at the end. The function ind2sub used in the above definitions returns the subscripts of matrix A for a given index, where the subscript of a matrix A in MATLAB identifies its elements based on row and columns indexing and the index is used to identify the elements of a matrix using integers only after linearizing it. The behavior of the ind2sub function that we formalized is presented in Table 1.

### Moving Sum

*MATLAB function: movsum* (https://www.mathworks.com/help/matlab/ref/movsum.html)

*Behavior:* The function movsum returns a matrix such that each of its elements is equal to the sum of the corresponding element of input matrix and its neighbors, where the neighborhood is defined by the second argument (sliding window). For example, for A = [1 2 3 4 5 6], and k = 3, movsum(A,k) = [3 6 9 12 15 11]. When the value of k is odd, the window is centered at the current index and for an even value, it is centered about the current and the previous indices. To pick the different number of elements in forward or backward direction movsum (A, [k_1_,k_2_]) is used, which computes the sum of the previous k_1_ elements, current element and the next k_2_ elements.

**Definition 13.** Moving Sum of the Matrix

⊦_*def*_ ∀(A:real^N^M) k. *movsum* A k = lambda i j.

          *if* |*k*|_2_ = 0 *then* \sum^{i+ (\frac{k}{2})}_{x = i- {\frac{k}{2}}} (*if* x = 0 ∨ x > M *then* 0 *else* A_xj_)

               *else* \sum^{i+ (\frac{k-1}{2})}_{x = i- {\frac{k-1}{2}}} (if x = 0 ∨ x > M then 0 else A_xj_)

⊦_*def*_ ∀(k:num^2^1) (A:real^N^M). *movsum_2* A k = lambda i j.

          \sum^{i+ (k_2)}_{x = i- {k_2}} (if x = 0 ∨ x > M *then* 0 *else* A_xj_)

### Moving average

*MATLAB function: movmean* (https://www.mathworks.com/help/matlab/ref/movmean.html)

*Behavior:* The function movmean returns the matrix with the local k point average of the elements of an input matrix A. It mainly adds the values of the elements within the given sliding window, as defined in the movsum function, and returns a matrix with their average values, that is, by dividing the sum value with the number of elements added.

**Definition 14.** Moving Average of the Matrix

⊦_def_ ∀(A:real^N^M) k. *movmean* A k = lambda i j.

        *if* |*k*|_2_ = 0 *then*

          *if* (1 ≤ i − \frac{k}{2}) ∧ (*i* + (\frac{k}{2} − 1) ≤ M) *then* \sum^{i+(\frac{k}{2}- 1) }_{x = i - \frac{k}{2}} \frac{A_{xj}{k}

          *else if ¬*(1 ≤ *i* − \frac{k}{2} ∧ (*i* + (\frac{k}{2} − 1) ≤ M) *then* \sum^{i+\frac{k}{2}- 1 }_{x = 1} \frac{A_{xj}{i+({{\frac{k}\over{2}}- 1) }

             *else if* (1 ≤ (*i* − \frac{k}{2}) ∧ *¬*(*i* + (\frac{k}{2} − 1) ≤ M) then \sum^{M}_{x = i - \frac{k}{2}} \frac{A_{xj}{(M - ({i-({{\frac{k}\over{2}}+ 1))) }

               *else* \sum^{M}_{x = 1} }{}(\frac{A_{xj}{M})

        *else if* (1 ≤ *i* − \frac{k-1}{2}) ∧ (*i* + (\frac{k-1}{2} − 1) ≤ M) then \sum^{i+(\frac{k-1}{2}-1)}_{x = i - \frac{k-1}{2}} \frac{A_{xj}{k}

          else if *¬*(1 ≤ *i* − \frac{k-1}{2} ∧ (*i* + \frac{k-1}{2} ≤ M) then \sum^{i+(\frac{k-1}{2}-1)}_{x=1} \frac{A_{xj}{(i+{\frac{{k-1}\over{2}})}

          *else if* (1 ≤ (*i* − \frac{k-1}{2}) ∧ *¬*(*i* + (\frac{k-1}{2}) ≤ M) then \sum^{M}_{x = i - \frac{k-1}{2}} \frac{A_{xj}{(M-(i-({{{k-1}\over{2}}}+1)))}

               *else* \sum^{M}_{x = 1} \big(\frac{A_{xj}{M}\big)

We modeled this behavior in HOL Light using the function movmean that accepts a matrix A and a number k and returns the desired value by using if-then-else statements to cover all the possible cases for inputs.

### Minimum

*MATLAB function: min* (https://www.mathworks.com/help/matlab/ref/min.html)

*Behavior:* Returns the Minimum Value in the Matrix

**Definition 15.** Minimum of the Matrix

⊦_*def*_ ∀y n. *min_vect* y 0 = min (y_1_) (y_2_) ∧

   *min_vect* y (n + 1) = min (*min_vect* y n) (y_*n*__+ 1_)

The function min_vect returns the minimum value of the vector y. To make the matrix input compatible, the function vectorize is used. The function vectorize is used to linearize the matrix to change it into a vector.

We also formally verified some of the classical mathematical properties for the formalized MATLAB functions. The verification of these properties, not only ensures the correctness of our definitions, but also facilitates the formal reasoning process about the systems that use the corresponding definitions. Some of these formally verified properties are briefly described below and more details and the full list of formally verified properties can be seen in Gauhar (2021).

**Theorem 1.** Multiplication with Concatenated Matrices

⊦_*thm*_ ∀A B C. A ∗ (horz_conct (B, C)) = horz_conct (A ∗ B, A ∗ C)

⊦_*thm*_ ∀A B C. (vert_conct (A, B)) ∗ C = vert_conct (A ∗ C, B ∗ C)

The above theorem provides an important property about the multiplication of the horizontal and vertical concatenation matrices. We start the proof process of Theorem 1 (horizontal concatenation) by rewriting with the definitions of horizontal concatenation (horz_conct in Table 1) and matrix multiplication (Definition 1) providing its components, that is, rows and column entries. Next, we used some properties of indexing along with arithmetic reasoning to conclude the proof of Theorem 1. We used the same kind of reasoning for the verification of the property about multiplication of the vertical concatenation matrices.

**Theorem 2.** Zero Matrix

⊦_*thm*_ ∀k. diag_matrix (vec 0) (k) = (mat 0)

This property guarantees the correctness of the diag_matrix function, which places the given vector as the diagonal of the given matrix. We start the verification of the above theorem by rewriting with the definitions of diag_matrix (Definition 10), vec (Definition 5) and mat (Definition 4). Next, we perform a case analysis on k along with some arithmetic reasoning to conclude with the proof of Theorem 2.

**Theorem 3.** Extracting Main Diagonal of Matrix

⊦_*thm*_ ∀k. diag_vect (mat k)(0)= vec k

The proof of Theorem 3 is based on the fact that, for any diagonal matrix having same entries in the main diagonal, the diag_vect returns the vector having elements equal to the main diagonal of the given matrix, given that *k* = 0. It requires rewriting with Definition 4, some basic properties of indexing along with some arithmetic reasoning.

**Theorem 4.** Cumulative Product of a Matrix

⊦_*thm*_ ∀(A:real^N^M) i j. (1 *≤* i *≤* N) ∧ (1 *≤* j *≤* N) ∧ (A_ij_ = 1) ⇒ cumprod_row A = A

The theorem states that for all-ones input matrix A, that is, an input matrix with all its entries equal to 1, the function cumprod_row returns the same matrix. The verification of Theorem 4 is mainly based on the definition cumprod_row in Table 1, some basic properties of indexing along with some arithmetic reasoning.

**Theorem 5.** Equivalence of Cumulative Product of a Matrix

⊦_*thm*_ ∀(A:real^N^M) i j. (1 *≤* i *≤* N) ∧ (1 *≤* j *≤* N) ∧ (A_ij_ = 1)

⇒ cumprod_row A = cumprod_col A

The above theorem provides an equivalence of the cumulative product along rows and column for all-ones input matrix A, that is, an input matrix with all its entries equal to 1. The verification of Theorem 5 is mainly based on definitions cumprod_row and cumprod_col in Table 1 and some indexing properties.

**Theorem 6.** Cumulative Sum of Zero Matrix

⊦_*thm*_ cumsum_row (mat 0) = cumsum_col (mat 0)

The proof of the above theorem is based on the fact that every element in a zero matrix is equal to zero and the property of summation of zero functions, which states that if a function is zero for a specific range then its sum in that range would also be zero.

**Theorem 7.** Equivalence of Moving Sum

⊦_*thm*_ ∀(A:real^N^M) (k:num^2^1)(n:num). (ODD n) ∧ (k_11_ = k_12_)

                     ⇒ movsum_2 A k = movsum A n

The theorem verifies the equivalence of the two variants of the function movsum and is based on arithmetic reasoning. The assumptions ensure that the length of sliding window is odd by using the function ODD and that both elements in the second argument of movsum_2 are equal.

**Theorem 8.** Sum of the Diagonal Matrix

⊦_*thm*_ ∀k. sum_row (mat k) = rowvector (vec k)

This property states that the sum of any diagonal matrix with all entries, which are equal to k, along its rows, is equal to a row vector, with all its elements equal to k. The proof of this theorem is primarily based on the definitions of the functions sum_row in Table 1, vec (Definition 5), rowvector (Definition 6), and mat (Definition 4) along with some arithmetic reasoning.

**Theorem 9.** Rotation of the Diagonal Matrix

⊦_*thm*_ *∀*n k. *if* k mod 2 = 0

      *then* rot90_matrix (mat n) (k) = mat (n)

      *else* rot90_matrix (mat n) (k) = flipud (mat n)

This theorem states that the counter-clockwise rotation of the diagonal matrix by odd multiples of 90° will result in the same matrix and for even multiples the function rot90_matrix flips the matrix upside down. The proof of this theorem is primarily based on the definitions of the functions mat (Definition 4) and rot90_matrix (Definition 9), induction on the variable k along with some arithmetic reasoning.

**Theorem 10.** Equivalence of Variants of mat_colon

⊦_*thm*_ ∀A u l k . (k = 1) ⇒ mat_colon_interval A l u k = mat_colon A l u

This theorem verifies the equivalence between the two variants of the colon indexing function when k = 1. The proof of this theorem is based on Definition 12 (the definitions of the functions mat_colon and mat_colon_interval) along with some arithmetic simplifications.

**Theorem 11.** Usage of mat_colon to Avoid Trailing Zeros

⊦_*thm*_ ∀(a:real^R^1) (b:real^Q^1) (n:num) (m:num) (k:num).

      (a = rowvector (vec k) ∧

      (1 ≤ n) ∧ (n ≤ R) ∧ (1 ≤ m) ∧ (m ≤ R) ∧

      (R ≤ Q) ∧ (Q = (n − m) + 1) ∧ (m ≤ n) ∧

      (b = rowvector (vec k)))

         ⇒ (mat_colon (b)(m)(n) + a) = (rowvector (vec (k + k)))

This theorem presents a way to eliminate the extra zeros, generated by the function mat_colon, by restricting the dimensions of the output to a desired number of elements and use these conditions in assumptions. The proof of the theorem is based on a lemma about indexing along with some arithmetic reasoning.

**Theorem 12.** Check the Element of mat_colon

⊦_*thm*_ ∀A u l. (l ≤ u) ⇒ (mat_colon (A)(l)(u))_1((*u*__−__*l*) + 1)_ = A_ind2sub__(*A*) (*u*)_

As mentioned above in Definition 12, mat_colon corresponds to the colon indexing in MATLAB, so this property guarantees its correctness by checking that ((u − l) + 1) element of the output must be equal to the *u*th element of input matrix A, assuming the value of l is less than or equal to u. The proof of the theorem is based on the definitions of the function mat_colon (Definition 12) and some reasoning on indexing along with arithmetic reasoning. Table 2 provides some additional properties of the formalized MATLAB matrix functions.

**Table 2:** Formally verified properties of matrix functions.

Property	Description
Diagonal of Zero Matrix	
⊦_*thm*_ ∀ k. diag_vect (mat 0) (k) = (vec 0)	The theorem states that with a zero matrix as input, any vector extracted via the diag_vect function would be a zero vector.
Placing Vector in Main Diagonal of Matrix	
⊦_*thm*_ ∀n. diag_matrix (vec n) (0) = mat n	This theorem states that when *k* = 0 then the diag_matrix for any input vector returns the diagonal matrix.
Flip Diagonal Matrix	
⊦_*thm*_ ∀n. fliplr (mat n) = flipud (mat n)	The proof of the theorem is primarily based on the arithmetic simplification on the row and column indices of the matrices
Element-wise Multiplication of Diagonal Matrix	
⊦_*thm*_ ∀n. array_mul (mat m) (mat n) = (mat m * n)	The proof of above theorem is primarily based on the definitions of the functions array_mul and mat along with some arithmetic reasoning
Power of the Diagonal Matrix	
⊦_*thm*_ ∀k p. (k ≠ 0) ⇒ matpow (mat k) = mat	The proof of this theorem is primarily based on the definitions of the functions mat and matpow along with some arithmetic reasoning about the power function
Rotation of the Zero Matrix	
⊦_*thm*_ ∀k. rot90_matrix (mat 0) (k) = mat (0)	The proof of this theorem is primarily based on the fact that rotation of the zero matrix returns the zero matrix

Just like the above-mentioned properties, we verified many other properties about our formalized MATLAB matrix functions and the details can be found in Gauhar (2021).

The formal proofs of Theorems 1–12 and the ones provided in Table 2 in our proposed formalization (https://doi.org/10.6084/m9.figshare.13992317.v1) of the matrix functions are mainly based on Definitions 1–15 and the ones presented in Table 1, indexing properties of the vectors and matrices along with some arithmetic reasoning. It is important to note that our HOL Light functions/definitions are generic, that is, having *N* dimensions of a vector and *N* × *M* dimensions of a matrix. On the other hand, in the MATLAB based analysis, these functions and theorems hold for specific values of vectors and matrices. The two main challenges incurred during the development of the proposed approach include:

- Developing a library of generic definitions for existing MATLAB functions. This was specially challenging in the case of functions that involved loops as we had to represent the corresponding behavior recursively in a formal manner.
- To facilitate the formal reasoning process as well as to ensure the correctness of our definitions, we had to figure out their behavioral properties (Theorems 1–12, Table 2) and interactively verify them in a theorem prover.

## Translator from matlab to hol light

**Figure 1:**
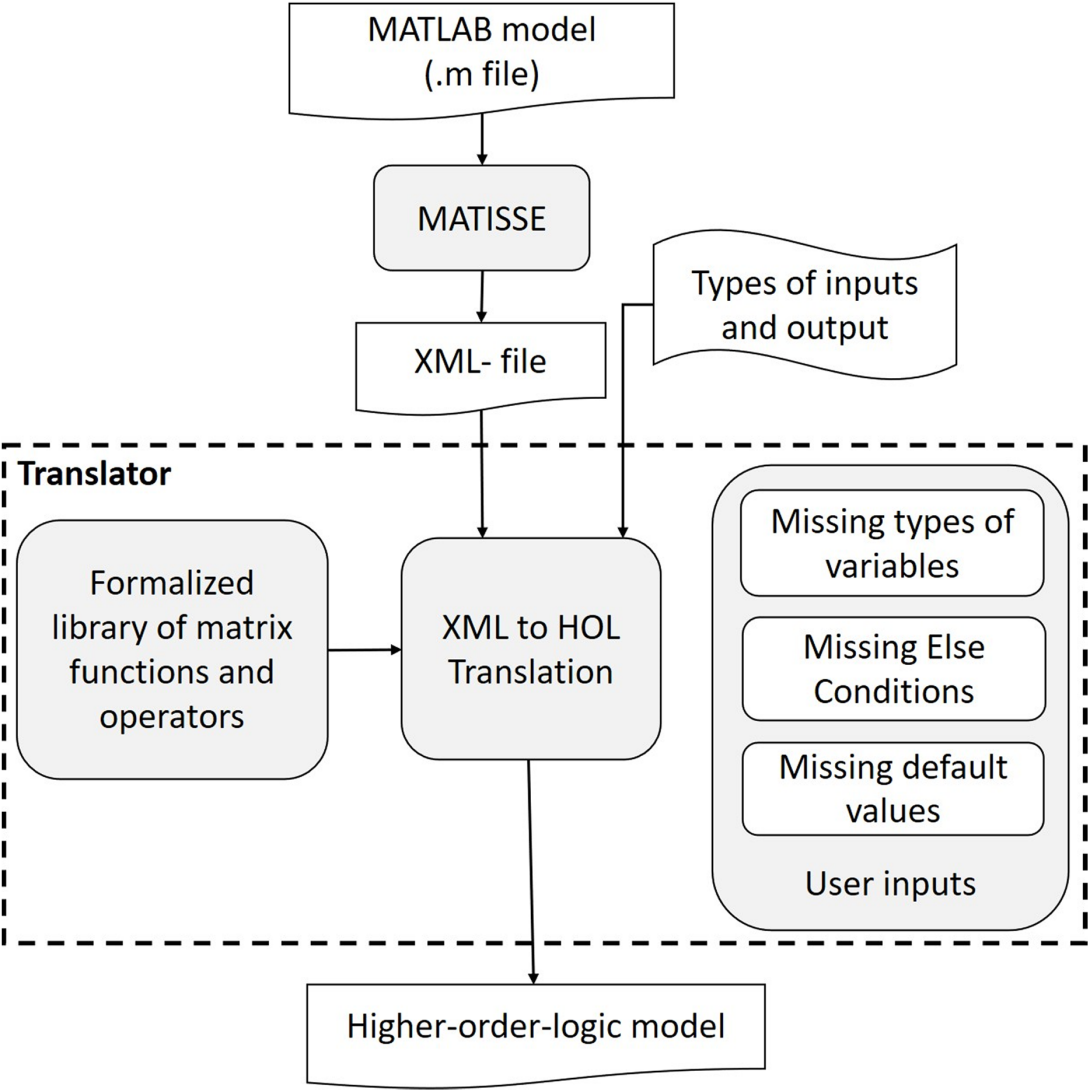
Overview of MATLAB to HOL light translation.

Using the formalization of the matrix functions described in the “Formalization Of Matrix Functions In Hol Light”, we developed a translator (https://doi.org/10.6084/m9.figshare.13992314.v1) for converting MATLAB models to the corresponding higher-order-logic models automatically. The overall architecture of the proposed framework is depicted in Fig. 1.

The translator works with compilable MATLAB models (m-files), that is, free of syntax problems, and uses the MATLAB functions that we have formalized. The translation process is broadly divided into two phases, that is, MATLAB model (*m*-file) to XML conversion and XML to HOL Light model conversion. The intermediate XML format is used because it provides a well-tagged parsed format that can be translated to HOL Light code in a straightforward manner. For the MATLAB to XML conversion, we use MATISSE (Bispo et al., 2013) a compiler that has been developed primarily for the conversion of MATLAB models to C code (Bispo et al., 2013). Although the compiler is not yet publicly available, it has an online demo version (http://specs.fe.up.pt/tools/matisse/) and we have made available a MATLAB-to-XML client that communicates with the online version^1^ 1The MATLAB -to- XML client can be downloaded at specs.fe.up.pt/tools/matlab-to-xml.jar..

The current work along with Bispo et al. (2013) can allow engineers to write a MATLAB model only to describe the desired system behavior. This model can then be used to simulate the design, to generate its formal model automatically, using the proposed translator, for formal verification, and to get the implementation in C code automatically using Bispo et al. (2013).

An important aspect of the proposed translator is to ensure the completeness of the model and for this purpose it has to deal with missing (i) data types, (ii) dimensions of matrices, (iii) values of variables and (iv) conditions. For example, the translator uses the same data types in the formal models that are specified in the corresponding MATLAB models. However, if a data type or dimension is missing then the translator uses the arbitrary data type or dimension. Similarly, MATLAB allows users to define models using an if-statement without an else statement, but the else part cannot be avoided in the corresponding formal model. In such scenarios, the proposed translator prompts the user to enter the action to be taken if the condition is not true.

Furthermore, MATLAB is an interpreted dynamic programing language that does not require variable declaration or type definition at compile time. Similarly, the MATLAB-to-XML converter does not distinguish between the constant multiplication and array multiplication because of the usage of the same operator (.∗). Similarly, there are many other operator overloadings that are commonly used in MATLAB. In these cases, the translator replaces the operators with the corresponding HOL Light functions/operators by inferring them based on the data types of the variables/inputs.

**Figure 2:**
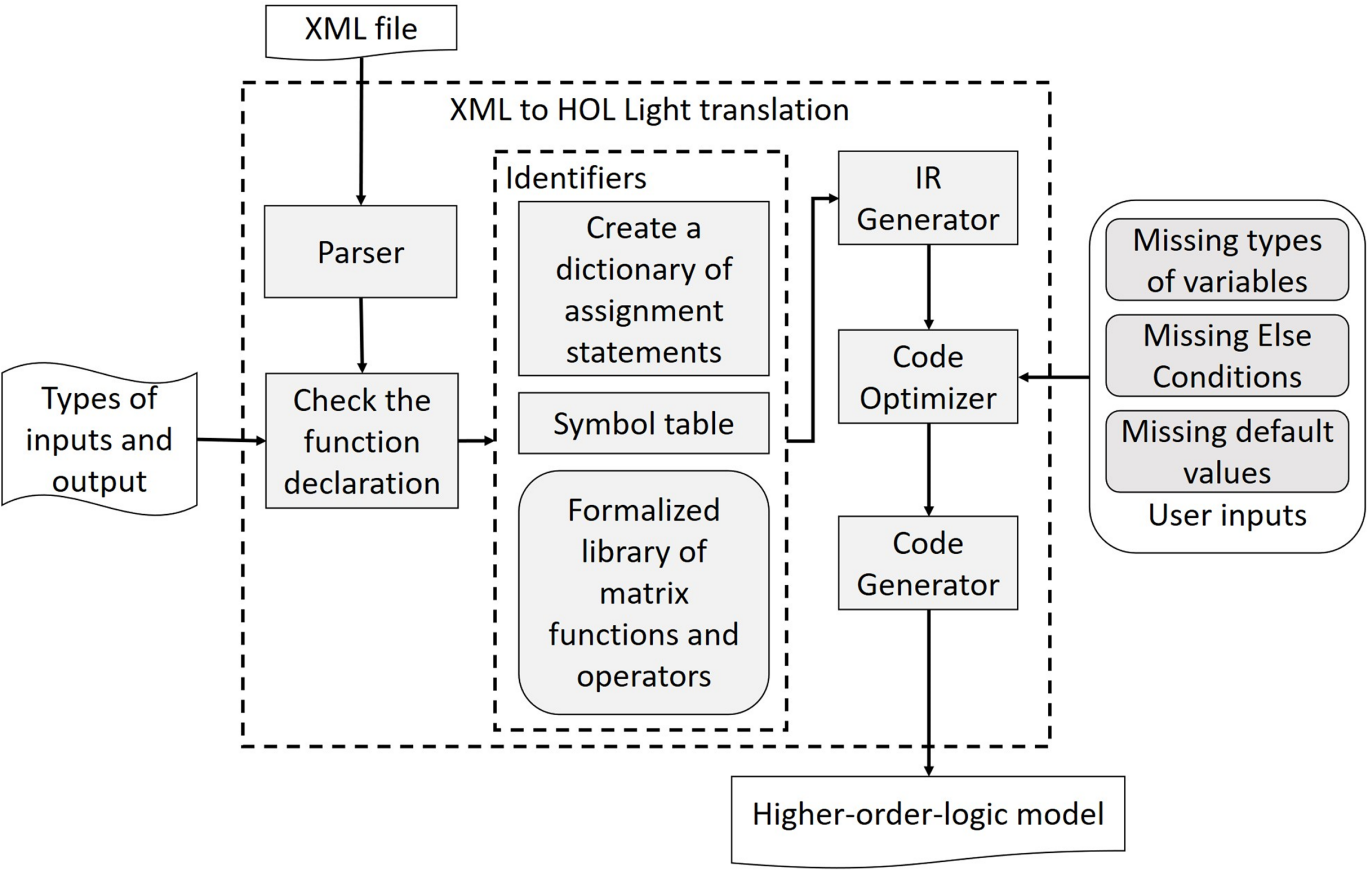
XML to HOL light translator’s architecture.

The proposed MATLAB code to HOL Light translator is depicted in Fig. 2. We first use the MATISSE compiler (http://specs.fe.up.pt/tools/matisse/) to output an XML file with an AST-based intermediate representation corresponding to the given MATLAB code. The XML file is then given to the XML parser to extract the content of tags and subtags along with their names. If the translator identifies an object with a function data type then the user is prompted to enter the types of its inputs and outputs. In case the user does not provide the types, then the input and output types are assumed to be arbitrary *M × N* matrices. Once the types of inputs and outputs are acquired, the translator constructs a symbol table for the identifiers along with a dictionary holding their values.

Both variables and functions of the MATLAB models are expressed with the identifier tag in XML representation. To avoid any ambiguity, the tables and dictionaries are formed by concurrent comparison of each identifier with the already stored names of MATLAB functions along with the formalized library presented in Section *Formalization of Matrix Functions in* HOL Light. Combining the information of symbol table, dictionary and our formalized library, the Intermediate Representation (IR) generator generates an IR of each statement depending on its type. The IR is chosen such that it captures all the information of the statement as well as can be easily used by the subsequent steps to generate the higher-order-logic model. Then, the code optimizer block revamps the output from IR generator according to the semantics of the target language and by taking some input from users, like missing data types and if-else conditions. Finally, the code generator develops the higher-order-logic model according to the syntax and semantics of the language used by the HOL Light theorem prover.

The translator has been developed to facilitate the formalization process but it has not been formally verified so there is always a chance that the translation process may introduce a bug in the formal model, like it might happen in the manual formalization process as well. However, since we formally verify the formal models in the next step there is a high probability of catching these kinds of translation bugs during the verification phase.

## Application: finite impulse response filter

Filters are widely used in Digital Signal Processing (DSP) applications to reduce or eliminate the unwanted components of the signal. Finite Impulse Response (FIR) filters find their applications in most of the DSP applications due to their inherent stability (Proakis & Manolakis, 2007).

**Figure 3:**
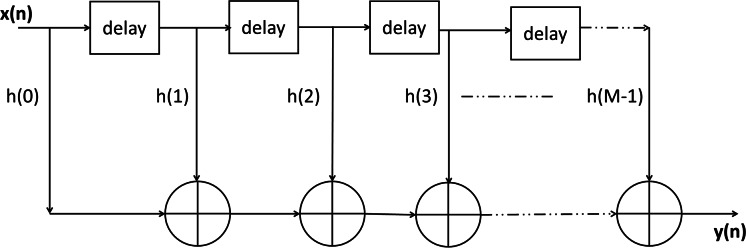
Finite impulse response (FIR) filter.

Finite Impulse Response filters provide a finite duration response when excited with a non-zero input. They are also called non-recursive filters due to the fact that the output at a particular instant is dependent on the past and present values of the input rather than the previous values of output. Mathematically the nth output of a M-tap/order FIR Filter (Lyons, 2011), as depicted in Fig. 3, can be expressed as:

(1) }{}y(n) = \sum^{M-1}_{k = 0} h(k) \ast x(n-k)

where *h*(*k*) is the impulse response or filter coefficients of the filter, *x*(*k*) is the input and *y*(*n*) represents the *n*th sample of output *y*.

A MATLAB model representing a simple FIR filter is expressed in Listing 1, where vector_1d and coef represent input and filter coefficients, respectively, and output is the output of the FIR filter.

**Listing 1.**
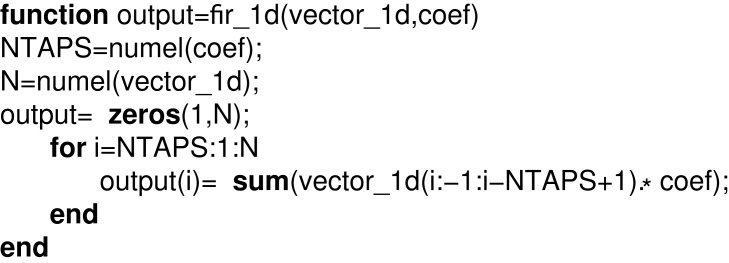
MATLAB model of 1D FIR filter.

As expressed in Eq. (1), the corresponding MATLAB model is formed by using the array multiplication, colon indexing of the matrix and summation functions. We have formalized all these functions and thus the translator formalized the behavior of this FIR filter using our formally defined functions array_mul, mat_colon, numel_mat, colon2 and sumrowvec as follows:

**Definition 16.** Formalization of 1D FIR Filter

⊦_*def*_ ∀(vector_1d:real^Q^P) (coef:real^R^1). *output* vector_1d coef = lambda i j.

 *if* colon2 j (numel_mat coef) (numel_mat vector_1d) *then*

  sumrowvec (array_mul (mat_colon vector_1d j (j - (numel_mat coef) + 1)) coef)

        *else* &0

The function output in the above definition returns the matrix representing the output of the filter. The input to the system includes input vector vector_1d and filter coefficients coef, respectively. Now based on our formalization, we can formally verify that if the impulse response is absolutely integrable and the input is bounded, then the system is Bounded-input-bounded-output (BIBO) stable for this filter. This can be expressed as the following theorem:

**Theorem 13.** BIBO Stability of the FIR Filter

⊦_*thm*_ ∀(vector_1d:real^Q^P) (coef:real^R^1).

  bounded (vector_1d) ∧ (\exists M.\;\sum^{N}_{x=1} |c_{1x}| \leq M) ⇒ bounded (output (vector_1d)(coef))

The distinguishing features of the above theorem include the fact that it is verified based on the inherent sound reasoning of the theorem proving approach, accompanying all required assumptions so it is guaranteed to be accurate, unlike the properties verified by other traditional analysis techniques. Moreover, the result is applicable to all universally quantified variables in the theorem, that is, vector_1d and coef, unlike the MATLAB based analysis, which holds for specific values of vectors and matrices. The formal reasoning about the correctness of the above theorem is primarily based on the formally verified theorems, presented in “Formalization of Matrix Functions in HOL Light” of this paper, and thus was very straightforward. This clearly indicates the usefulness of the proposed approach for the formal analysis of MATLAB models involving matrices.

## Conclusions

This article presented a higher-order-logic formalization of some of the commonly used matrix functions of MATLAB. The formalization has been carried out by using the multivariate calculus theory of HOL Light. This formalization facilitates the development of formal models for MATLAB models, expressed as *m*-code. We have also developed an automatic translator to convert MATLAB models to their corresponding higher-order-logic formalization automatically. This formal model can then be used to formally verify its desired characteristics in HOL Light. The formally verified properties of our formalized functions in HOL Light greatly aid in the formal reasoning process of the corresponding system properties. For illustration purposes, we presented a formal analysis of a FIR filter and the modeling and analysis was found to be a very straightforward one, thanks to our formal definitions and theorems.

Currently our formalization supports a subset of MATLAB functions and we are planning to extend the proposed library by formalizing additional functions, such as matrix_inverse and convolution and thus cater for the formal analysis of more complex applications. In particular, the convolution function is widely used for performing the formal analysis of many continuous and discrete time systems, such as, digital image processing filters (Solomon & Breckon, 2011) and neural networks (Niepert, Ahmed & Kutzkov, 2016). Automating some parts of the verification process by writing specialized proof tactics is another worth exploring research direction for this work.

## Additional Information and Declarations

### Data Availability

The following information was supplied regarding data availability: Data are available at Figshare: - Translator: Rashid, Adnan (2021): Translator. figshare. Software. DOI 10.6084/m9.figshare.13992314.v1 - Translator Manual: Rashid, Adnan (2021): Translator Manual. figshare. Dataset. DOI 10.6084/m9.figshare.13992320.v1. HOL Light Code is also available at Figshare: Rashid, Adnan (2021): HOL Light Code. figshare. Dataset. DOI 10.6084/m9.figshare.13992317.v1.
